# Quantitative whole‐body magnetic resonance imaging in children with Pompe disease: Clinical tools to evaluate severity of muscle disease

**DOI:** 10.1002/jmd2.12174

**Published:** 2020-10-14

**Authors:** Samuela A. Fernandes, Aleena A. Khan, Tracy Boggs, Michael Bowling, Stephanie Austin, Mihaela Stefanescu, Laura Case, Priya S. Kishnani

**Affiliations:** ^1^ Division of Medical Genetics, Department of Pediatrics Duke University School of Medicine Durham North Carolina USA; ^2^ Division of Physical Therapy, Department of Community and Family Medicine Duke University School of Medicine Durham North Carolina USA; ^3^ Multi‐Dimensional Image Processing Laboratory, Department of Radiology Duke University School of Medicine Durham North Carolina USA; ^4^ Doctor of Physical Therapy Division, Department of Orthopedics Duke University School of Medicine Durham North Carolina USA

**Keywords:** infantile Pompe disease, IPD, late‐onset Pompe disease, LOPD, muscle function testing, muscle strength testing, newborn screening, Pompe disease, WBMRI, whole‐body magnetic resonance imaging

## Abstract

**Objective:**

Since the introduction of enzyme replacement therapy (ERT) with alglucosidase alfa, there has been increased survival in patients with Pompe disease. It is essential to characterize and quantify the burden of disease in these patients. Here, we report a measure of muscle fat infiltration in children with infantile and pediatric late‐onset Pompe disease (IPD and LOPD, respectively) to better understand the extent of muscle involvement.

**Methods:**

Eleven pediatric patients with Pompe disease (five IPD, six LOPD), ages 7‐17 years, received whole‐body magnetic resonance imaging (WBMRI), muscle strength testing using the modified Medical Research Council (mMRC) scale, functional assessment using gait, stairs, gowers, chair (GSGC), and urine glucose tetrasaccharide (Glc_4_) testing. The intramuscular fat seen on WBMRI was quantified using proton density fat fraction (PDFF) and correlated to appropriate muscle strength and functional tests, and urine Glc_4_.

**Results:**

Patients with IPD, although younger, had higher mean PDFF values than LOPD patients (11.61% vs 8.52%). Significant correlation existed between PDFF and the GSGC assessment (*r* = .9273, *P* = .0003). Moderate correlation existed between PDFF and mMRC (*r* = −.667, *P* = .0831), and PDFF and urine Glc_4_ (*r* = .6121, *P* = .0667). Anterior tibialis was in the top quartile of muscle involvement for patients with LOPD.

**Conclusion:**

In the past, physical therapy assessments alone have been used to track disease progression. Here, we show the clinical utility of WBMRI in quantifying muscle involvement in children with Pompe disease, especially regarding the novel involvement of anterior tibialis in children with LOPD, to better assess baseline muscle burden and mapping disease progression in children treated with ERT.

**Abbreviations**
Glc_4_: glucose tetrasaccharide
GSGC: gait, stairs, gower, chair
IPD: infantile‐onset Pompe disease
LOPD: late‐onset Pompe disease
mMRC: modified Medical Research Council
PDFF: proton density fat fraction
WBMRI: whole‐body magnetic resonance imaging

**Synposis**

This study demonstrates the clinical utility of whole‐body MRI in quantifying disease burden in children with Pompe disease in order to better guide treatment, especially as it relates to the novel finding of anterior tibialis involvement in children with LOPD.

## 1 INTRODUCTION

Pompe disease or glycogen storage disease type II (GSD II, OMIM 232300) is a rare autosomal recessive neuromuscular disease due to a deficiency in the lysosomal enzyme acid α‐glucosidase (GAA).^1^ In the absence of GAA, there is accumulation of glycogen within lysosomes, lysosomal rupture, and tissue damage in multiple organs. This mechanism gives rise to a variety of symptoms, including proximal skeletal muscle weakness, fatigue, respiratory insufficiency, and cardiomyopathy.^2^ Within muscular tissue this results in damage, fibrosis, and eventual intramuscular fat accumulation.^3^

Patients who develop hypertrophic cardiomyopathy within the first year of life are categorized into infantile‐onset Pompe disease (IPD).^4^ All other patients are classified as late‐onset Pompe disease (LOPD) and may present at any age (infancy to sixth decade of life). Regardless of the classification, the symptoms in Pompe Disease fall on a spectrum, with increased severity in patients who present at a young age. Timely diagnosis and initiation of enzyme replacement therapy (ERT) is essential to prevent irreversible tissue damage and slow the progression of disease. This makes diagnosis and disease monitoring of great importance.

Many modalities exist to monitor patients with neuromuscular diseases such as Pompe: physical therapy (PT), pulmonary function tests, biochemical markers, patient assessments of quality of life, and in some instances serial muscle biopsies.^5^, ^6^, ^7^, ^8^ Physical therapy assessment of muscle strength and function is currently one of the most accepted methods to assess functional status. These assessments have limitations, creating a need for quantitative, noninvasive methods for monitoring disease and treatment response. Whole‐body muscle MRI (WBMRI) has unique utility in this regard.^9^, ^10^, ^11^, ^12^

WBMRI produces images where intramuscular fat percentage can be quantified through a parameter known as proton density fat fraction (PDFF).^12^ Our group recently demonstrated that PDFF is a sensitive indicator of disease status as performance across various PT assessments correlated highly with PDFF.^13^ This enables the use of PDFF to monitor patients and evaluate the response to therapy. However, whether PDFF extracted from WBMRI of pediatric Pompe patients (both IPD and LOPD) is similarly useful remains unknown.

In this study, we explore the utility of WBMRI in a pediatric Pompe cohort. We evaluate the extent and differences of intramuscular fat involvement between IPD, pediatric LOPD, and previously published adult LOPD patients. As previously, we investigate the relationship of muscle fat infiltration with PT assessments and laboratory biomarkers. To the best of our knowledge, this is the first report in the pediatric Pompe population that utilizes WBMRI to quantify intramuscular fat infiltration and correlate these values to PT assessments, in order to assess the clinical utility of quantitative WBMRI in pediatric Pompe Disease.

## 2 METHODS

### 2.1 Patients

Patients under the age of 18 years with a confirmed genetic diagnosis of Pompe disease were enrolled in the study under an IRB‐approved protocol (Pro00047132). Informed consent was obtained from a parent or legal guardian. Data was collected retrospectively through chart review. Individuals who were ventilator dependent, could not safely lie supine, or required sedation to undergo WBMRI were excluded from the study. Patients received PT assessments and lab evaluation within 13 months of each WBMRI scan.

### 2.2 Muscle strength and functional testing

PT assessments were performed by four physical therapists who had high inter‐rater reliability, and were experienced in neuromuscular pathology. Manual muscle testing was evaluated via the modified Medical Research Council (mMRC) scale. Strength testing was performed for the following movements: hip flexion, hip extension (with flexed knee and with extended knee), hip abduction, hip adduction, knee extension, knee flexion, ankle dorsiflexion. The muscles associated with each movement are represented in Table S1a. Each movement is graded on a scale from 0 (no movement) to 5 (full movement) with additional values (0, 1, 2−, 2, 2+, 3−, 3, 3+, 4−, 4, 4+, 5−, 5) that represented intermediate performance. As previously published, this 0 to 5 scale was converted to a 0 to 12‐point scale to organize scoring in a linear manner.^13^

Muscle function was evaluated with the gait, stairs, gowers, chair (GSGC) assessment which has been previously validated in LOPD and is clinically utilized in IPD.^14^ The activities include: Gait (walking 10 m at a comfortable, self‐selected speed); stairs (climbing four steps as safely fast as possible without hand support); gowers (transitioning from supine on the ground to standing as safely fast as possible without hand support); and chair (transitioning from sitting in a chair to standing as safely fast as possible without hand support). Trained physical therapists assign a score from 4 (normal) to 27 (severe/unable to complete task), based on qualitative assessment of the patient's performance of the assigned tasks. The four component activities test compound functions of many muscles, as represented in Table S1b.

### 2.3 Whole‐body magnetic resonance imaging

Each patient received a WBMRI scan on the same 3 T MRI system. Imaging was performed in the supine position, and axial images were captured, as previously reported by Khan et al.^13^ On average, scans took 60‐90 minutes, capturing 11 sequences per anatomical location; each one was evaluated for anatomy, pathology and artifact.

**FIGURE 1:**
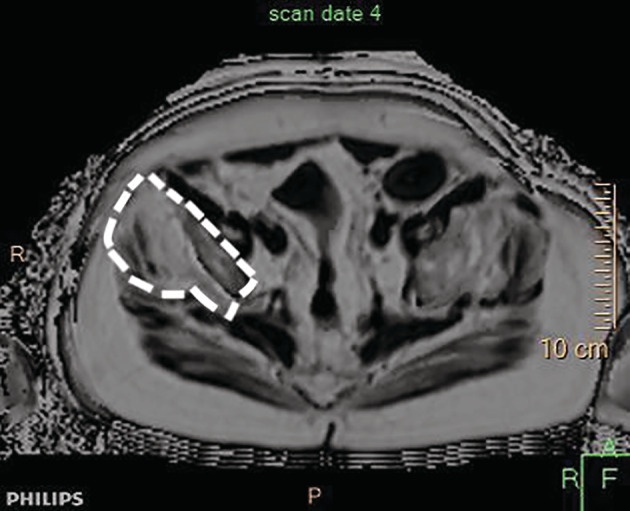
Axial section of thigh muscles in an LOPD patient. Hypointense areas are normal and hyperintense areas demonstrate muscle fat infiltration. ROI technique is demonstrated. LOPD, late‐onset Pompe disease; ROI, region of interest [Correction added on 21 October 2020, after first online publication: Figure 1 was incorrect and has been corrected in this version.]

Fat seen on WBMRI was quantified using the PDFF technique where a region of interest (ROI) was delineated. An example of this technique is shown in Figure 1. A highly trained radiologic technician outlined the ROI, and a radiologist with experience verified results. The imaged muscles included: rhomboids, trapezius, serratus anterior, thoracic and lumbar extensors, gluteus maximus, gluteus medius, iliopsoas, rectus femoris, vastus medialis, intermedius and lateralis, adductor magnus, longus and brevis, hamstrings and anterior tibialis.

The average fat fraction observed in healthy pediatric controls is known to be around 5%, varying slightly between muscles.^15^ This value should be considered when interpreting the PDFF values presented in our manuscript.

### 2.4 Analysis and statistics

The mean PDFF of muscle groups involved in movements of strength and function testing (Table S1) were calculated and correlated to the mMRC and GSGC scores. Additionally, we correlated mean PDFF of all muscles to urinary glucose tetrasaccharide (Glc_4_), a biomarker representing glycogen accumulation in muscle.

Descriptive statistics are reported in three cohorts: all patients (n = 11), IPD only (n = 5), and LOPD only (n = 6). Data were analyzed to assess and compare which muscles have most fat infiltration in each of these cohorts. Correlation using Spearman's test for nonparametric populations was performed. We utilized *r* > .6 to indicate a strong correlation, and a *P*‐value of <.05 to indicate statistical significance.^16^ All statistical analyses were performed on GraphPad Prism 8 (GraphPad Software, San Diego, California; 2018).

## 3 RESULTS

### 3.1 Patients

Eleven confirmed pediatric Pompe patients (six males, five females) met inclusion criteria and were evaluated for this study. There were five IPD and six LOPD patients. Age at the start of the study based on the date of WBMRI ranged between 7 and 17 years with a median age of 12 years; IPD patients had a median age of 12 years (range 7‐16 years), while LOPD patients had a median age of 13 years (9‐17 years). All patients were on ERT at the time of the study. Two IPD patients were not independently ambulatory: patient 3 utilized a walker and patient 4 was wheelchair bound. Table S2 represents patients' demographic information, ERT doses at the time of WBMRI and pathogenic variants.

Eight out of 11 patients had mMRC testing; three IPD patients did not have mMRC (patients 2, 3, 5). Ten out of 11 patients had complete GSGC testing; patient 4 is wheelchair bound and was unable perform any of the components of the GSGC earning him the poorest score, and patient 5 did not receive formal PT testing. Ten out of 11 patients have a urine Glc_4_ level.

### 3.2 PDFF differences between IPD and LOPD

**FIGURE 2:**
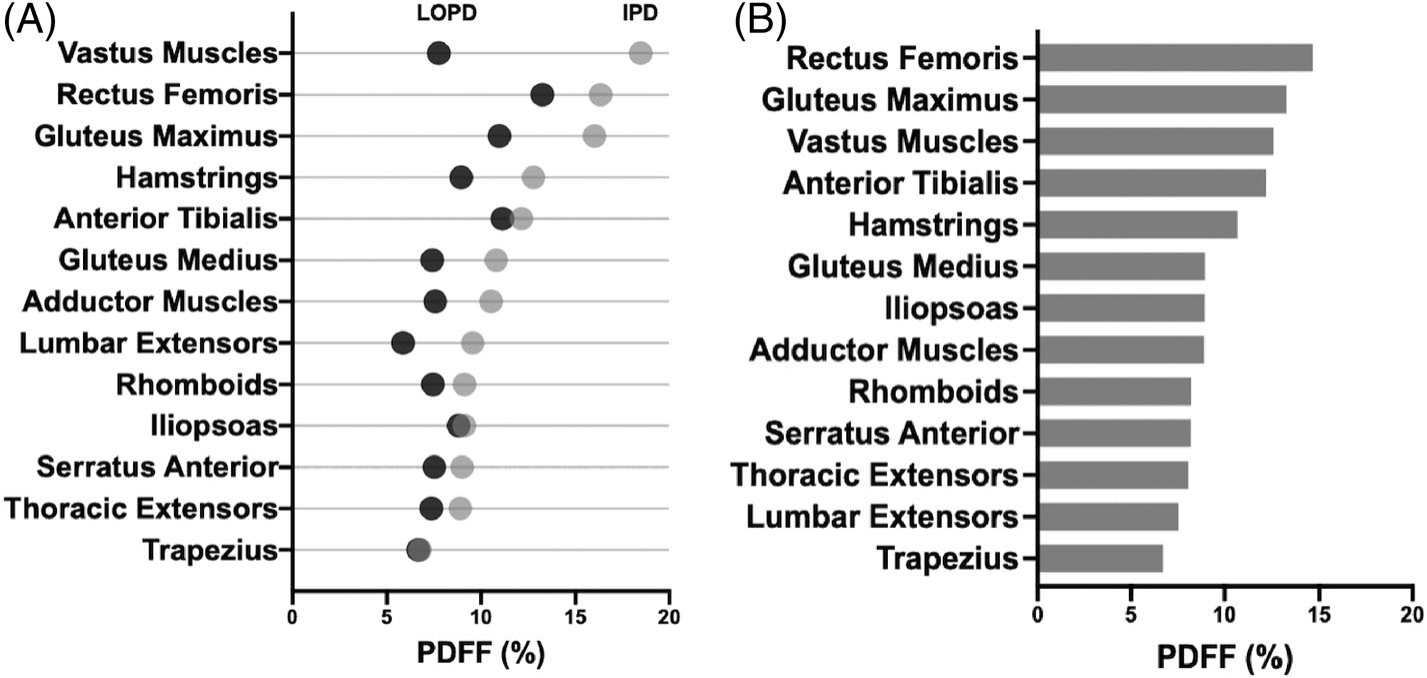
A, Comparison of muscle specific PDFF values between IPD cohort (patients 1‐5), and pediatric LOPD cohort (patients 6‐11), ordered from most involved muscles in IPD to least involved muscles in IPD. B, Muscle specific breakdown of PDFF values across all 11 patients, ordered from most to least involved. IPD, infantile Pompe disease; LOPD, late‐onset Pompe disease; PDFF, proton density fat fraction [Correction added on 21 October 2020, after first online publication: Figure 2 was incorrect and has been corrected in this version.]

In the IPD cohort, the mean overall PDFF for all muscles was 11.61% whereas in LOPD cohort, the mean overall PDFF was 8.52%. In the IPD cohort, the most involved muscles were the vastus muscles (18.48%), rectus femoris (16.34%), and gluteus maximus (16.02%). Anterior tibialis (13.53%) was the fourth most involved muscle. In the LOPD cohort, the most involved muscles were rectus femoris (13.25%), anterior tibialis (11.14%), and gluteus maximus (10.97%). Figure 2A represents a comparison of IPD and LOPD muscle involvement, and the PDFF values are available in Table S3.

### 3.3 PDFF findings in the combined cohort

In the 11 patients evaluated, the mean PDFF for all muscles was 9.92%. The highest PDFF values were seen in the rectus femoris (14.66%), gluteus maximus (13.26%), and vastus muscles (12.63%). The mean PDFF values for each muscle measured are represented in Figure 2B. By quartile, the most involved patients were as follow: patient 4 (IPD, 24.32%), patient 2 (IPD, 11.57%), and patient 3 (IPD, 10.62%); the least involved patients were patient 7 (LOPD, 5.88%), patient 5 (IPD, 5.82%), patient 1 (IPD, 5.71%). Six of 11 patients had the rectus femoris as the most involved muscle (patients 1, 2, 5, 7, 10, 11). Three of 11 had the anterior tibialis as the most involved muscle (patients 3, 8, 9). One of 11 had the gluteus medius as the most involved muscle (patient 6), and another (patient 4) had highest involvement in the vastus muscles.

### 3.4 PDFF correlation with muscle strength testing

There is evidence of a possible association between the mean mMRC score of all tested muscles and mean PDFF (*r* = −.667, *P* = .0831. Figure S1A). The most suggestive trends were seen with knee extension testing (*r* = −.7066, *P* = .0595) and knee flexion testing (*r* = −.6587, *P* = .0881. Figures S1A and S1C). Table 1 summarizes the correlation of mMRC scores and their corresponding PDFF values of muscles.

**TABLE 1:** Table of the correlation coefficients and significance of mMRC testing, GSGC testing, and lab values, compared to PDFF values

	*r*	*P*
mMRC		
mMRC vs mean PDFF selected muscles	−.667	.0831
Hip flexion vs iliopsoas + rectus femoris	−.4636	.2604
Hip extension with flexed knee vs gluteus maximus	−.6	.4167
Hip extension with extended knee vs gluteus maximus + hamstrings	−.5389	.1769
Hip abductor vs gluteus medius	−.4762	.2431
Hip adductor vs adductor muscles	−.5422	.1706
Knee extension vs vastus muscles	−.7066	.0595
Knee flexion vs hamstrings	−.6587	.0881
Ankle dorsiflexion vs anterior tibialis	−.4671	.2456
GSGC		
GSGC vs mean PDFF of selected muscles	.9273	.0003
Gait (m/s) vs mean PDFF selected muscles	−.8	.0138
Time to climb 4 stairs (s) vs mean PDFF selected muscles	.5167	.1618
Gower (s) vs mean PDFF selected muscles	.6667	.0589
Time sitting to standing (s) vs mean PDFF selected muscles	.3333	.4279
Labs		
CK	.05	.9116
AST	−.25	.5206
ALT	.06667	.8801
Glc_4_	.6121	.0667

*Note:* PDFF values used for mMRC and GSGC testing were based on muscles involved in the actions of the testing; while PDFF values for the lab values were patients' overall mean PDFF. Glc_4_ range: >3 mmol/mol creatinine.

Abbreviations: ALT, alanine aminotransferase; AST, aspartate aminotransferase; CK, creatinine kinase; Glc_4_, glucose tetrasaccharide; GSGC, gait, stairs, gowers, chair; mMRC, modified manual research council; PDFF, proton density fat fraction.

### 3.5 PDFF correlation with functional testing

There was a very strong correlation (*r* = .9273, *P* = .0003) of the GSGC score with PDFF (Figure S2A). The correlation between the individual components of the GSGC assessment and their relevant muscle PDFF values is summarized in Table 1. A strong and significant PDFF correlation was seen with gait speed (*r* = −.8, *P* = .0138. Figure S2B), and a moderate correlation was seen with standing from supine (Gower) assessment (*r* = .6667, *P* = .0589. Figure S2C).

### 3.6 PDFF correlation with lab values

A moderately significant correlation was found with urine Glc_4_ (*r* = .6121, *P* = .0667). Range for normal Glc_4_ in patients older than 3 years is 3.0 mmol/mol creatinine.

## 4 DISCUSSION

In this study, we found that, although younger on average, the IPD cohort had higher PDFF values than the LOPD cohort. This finding is consistent with what is known about severity of the IPD phenotype. However, the muscles most commonly involved, and the difference in PDFF between specific muscles in the IPD and LOPD cohorts varied. The involvement of the rectus, vastus, and gluteus muscles in Pompe disease, both LOPD and IPD, has previously been established.^12^, ^13^, ^17^, ^18^ However, the level of involvement of the anterior tibialis in the LOPD cohort is of interest and potentially novel.

**FIGURE 3:**
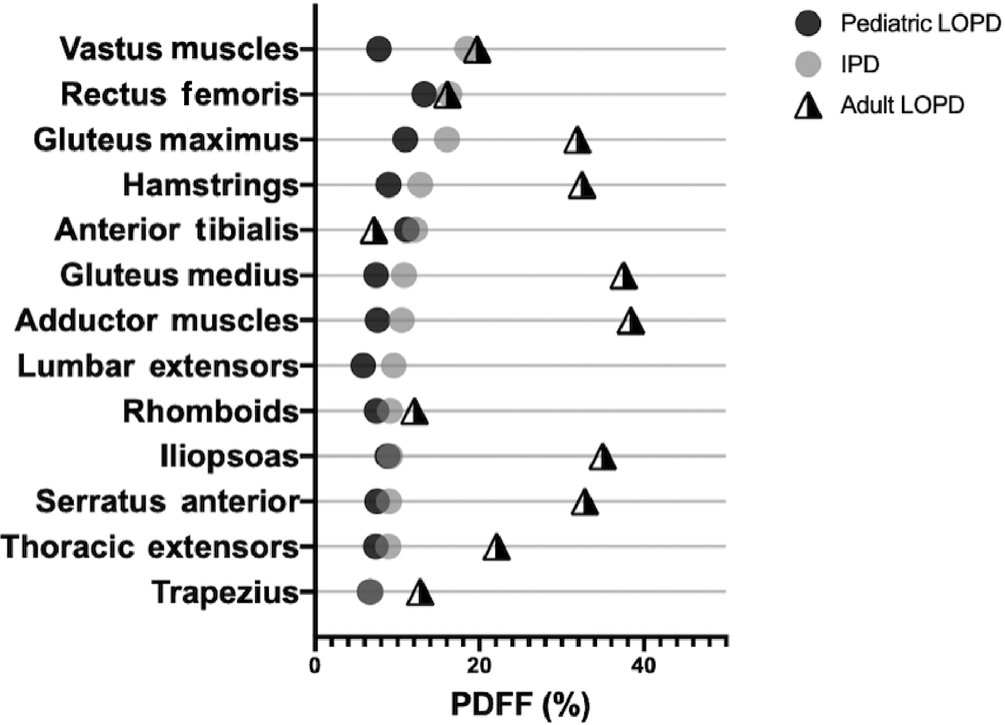
Muscle specific PDFF values for the previously published adult LOPD cohort and the current pediatric LOPD cohort. This demonstrates differing muscle involvement patterns between the two cohorts. Anterior tibialis involvement in pediatric LOPD cohort is novel.^13^ LOPD, late‐onset Pompe disease; IPD, infantile Pompe disease; PDFF, proton density fat fraction

Anterior tibialis weakness is clinically seen in IPD patients and has been previously established in the literature.^12^, ^18^ However, clinical involvement in LOPD patients has not been demonstrated clearly. Montagnese et al demonstrate a small minority of adult LOPD patients with anterior tibialis involvement and Khan et al showed that in her adult LOPD cohort, the anterior tibialis was the least involved muscle of those studied.^13^, ^19^ In our study, the anterior tibialis is one of the most involved muscles in the pediatric LOPD cohort. This difference between pediatric LOPD and adult LOPD patients is certainly interesting. In the current study, PDFF values of the anterior tibialis are similar for the IPD and LOPD cohort. This pattern suggests that early‐onset LOPD may resemble IPD more closely than adult LOPD. Figure 3 demonstrates a comparison of muscle involvement between the already published adult LOPD cohort in Khan et al and the pediatric LOPD cohort reported in this study. This raises the question of whether early anterior tibialis involvement, especially in children with LOPD, could indicate more severe disease.

We were also interested in the relationship of the biomarker Glc_4_ with PDFF values. A product of glycogen breakdown, urine Glc_4_ is a specific biomarker for muscle injury due to glycogen accumulation. There was a moderate correlation between mean PDFF and Glc_4_. Patients with lesser extent of muscle involvement (based on PDFF) had low Glc_4_ values, as expected. Patients with high PDFF had varying Glc_4_ values, with our most involved patient (patient 4, PDFF 24.32%) having a moderate Glc_4_ value of 12.1 mmol, while our second most involved patient (patient 2, PDFF 11.58%) having the highest Glc_4_ value of 43.7 mmol. These results somewhat support the idea put forth by Khan et al: perhaps severe disease states progress to a “burn‐out” point where urine Glc_4_ values begin to decrease as a result of muscle atrophy rather than lack of glycogen accumulation.^13^ In any case, Glc_4_ values should always be interpreted within the clinical context. Our data provide support that urine Glc_4_ levels are a helpful tool for monitoring disease progression in patients with Pompe disease.^20^

WBMRI has the potential to visually identify muscle fat infiltration before clinically apparent muscle dysfunction, allowing us to pre‐emptively treat patients. Owing to its quantitative nature, the PDFF could also help guide providers in monitoring both disease progression and response to treatment. Results of our study demonstrate that mean PDFF values correlate to GSGC and mMRC scores. Overall GSGC and mMRC correlated better to PDFF values compared to individual components of PT assessments. Similarly, gait speed and PDFF exhibited strong correlation. As reported previously, our findings suggest that compound movements utilizing a variety of muscles offer a better understanding of muscle disease and weakness on exam.^13^

These close correlations show that WBMRI can accurately predict functional status as ascertained by PT assessments. There is a role for serial WBMRI, especially the quantitative PDFF technique to monitor disease progression and response to treatment. The quantitative nature of WBMRI and the ability to trend muscle fat infiltration could better guide physicians trying to determine an appropriate time to begin therapy in asymptomatic LOPD patients, or when to increase ERT dosage in IPD or LOPD. In addition, WBMRI has the potential to give insight into specific muscle fat infiltration, as opposed to PT assessments which give an overall impression of functional status rather than specific muscle involvement. However, it is important to note that these correlations do demonstrate that in communities where WBMRI is not readily available, PT assessments, such as GSGC and mMRC may be used to grossly monitor general disease progression.

With the recent addition of Pompe to the Recommended Uniform Screening Panel for Newborn Screening (NBS), many LOPD patients are now diagnosed prior to onset of symptoms. Although clear guidelines are established for the immediate initiation of ERT in IPD, there is significant challenge in deciding when to begin therapy in LOPD. In the past, ERT has been initiated in LOPD patients when they begin to exhibit symptoms; however, it is likely that there is already significant tissue damage by that point.^21^ Prodromal symptoms such as mild hypotonia, delayed attainment of gross motor milestones, easy fatigability may be overlooked, and as clinicians, we may be waiting too long before initiating therapy.^21^ With clinical evidence indicating early initiation of ERT in LOPD patients yields better outcomes, diagnostic tools such as WBMRI offer promise.^22^

Here we report preliminary findings of relationships between muscle strength and function testing, mean PDFF values, and possible differences between IPD and LOPD. However, as the sample size is 11 patients, this is not a large enough cohort to draw generalizable conclusions. In addition, though our data showed clear trends, the small sample size contributed to the fact that many of our results were not statistically significant. Also, due to the retrospective nature of the study not all patients received the same testing within range of their WBMRI. Lastly, our data excludes patients who could not tolerate a WBMRI due to ventilatory requirements or very young patients who would require sedation for scans. Larger, longitudinal studies or perhaps prospective studies are required to fully characterize the possible relationships presented here. Future studies should look to assess younger patients and those requiring ventilatory support.

## 5 CONCLUSION

WBMRI is potentially a new valuable means of quantifying muscle fat infiltration and muscle disease status in children with Pompe. Muscle strength testing using mMRC and functional PT assessments such as GSGC show excellent correlation with muscle fat fraction, and may be used at centers where WBMRI is not available. We demonstrated that IPD patients have intramuscular fat involvement at a younger age than pediatric LOPD patients. IPD and pediatric LOPD cohorts share similar patterns of muscle involvement, specifically involvement of the anterior tibialis, especially when compared to involvement in adult LOPD patients, suggesting earlier involvement of this muscle in patients with more disease severity. With regard to future directions, there is a role for WBMRI to determine when to begin or increase dose of ERT, to monitor disease progression, and evaluate response to treatment.

## CONFLICT OF INTEREST

Priya S. Kishnani has received research/grant support from Sanofi Genzyme, Valerion Therapeutics, and Amicus Therapeutics; consulting fees and honoraria from Sanofi Genzyme, Amicus Therapeutics, Vertex Pharmaceuticals and Asklepios Biopharmaceutical, Inc (AskBio). She is a member of the Pompe and Gaucher Disease Registry Advisory Board for Sanofi Genzyme, Amicus Therapeutics, and Baebies; and has equity in Actus Therapeutics, which is developing gene therapy for Pompe disease.

## GUARANTOR AUTHOR

Priya S. Kishnani.

## INITIAL MANUSCRIPT DRAFT WRITTEN BY

Samuela A. Fernandes and Aleena A. Khan.

## CONCEPTUALIZATION/DESIGN

Laura Case, Stephanie Austin, Mihaela Stefanescu, and Priya Kishnani.

## METHODOLOGY OF STUDY

Laura Case, Stephanie Austin, Mihaela Stefanescu, Priya Kishnani Samuela Fernandes, and Aleena Khan.

## INVESTIGATION

Priya Kishnani, Laura Case, Michael Bowling, Samuela Fernandes, and Aleena Khan.

## SUPERVISION/OVERSIGHT

Priya Kishnani and Stephanie Austin.

## FUNDING ACQUISITION

Priya Kishnani and Stephanie Austin.

## DATA CURATION

Samuela Fernandes, Aleena Khan, Stephanie Austin, and Mihaela Stefanescu.

## FORMAL ANALYSIS

Samuela Fernandes and Aleena Khan.

## RESOURCES

Laura Case, Michael Bowling, and Tracy Boggs.

## Supporting information


**Figure S1**: A‐C (left to right): Graphical representations of the PDFF values for each patients' mMRC score. (A) plots the overall average mMRC based on a mean of the distinct strength testing; while (B) and (C) are the mMRC scores of strength testing around a specific joint. PDFF values were calculated based on the muscles involved in the actions performed. PDFF: proton density fat fraction, mMRC: modified manual research council

**Figure S2**: A‐C (left to right): Graphical representations of the PDFF values for each patients' GSGC score. (A) plots the overall average GSGC score; while (B) and (C) are the timed gait testing (meters/second) and gower testing (seconds). PDFF values were calculated based on the muscles involved in the actions performed. PDFF: proton density fat fraction, mMRC: modified manual research council, GSGC: gait, stairs, gower, chair

**Table S1**: (a) Muscles involved in manual muscle testing conducted in the study. (b) Muscles involved in the gait, stairs, gowers, chairs assessment used in this study.
**Table S2**: Table describing the demographic information of our subjects.
**Table S3**: Mean PDFF values of specific muscles, IPD and LOPD.
