# Supplementary figures and images for: Quantitative whole‐body magnetic resonance imaging in children with Pompe disease: Clinical tools to evaluate severity of muscle disease
Source: JIMD Rep. 2020 Oct 14;57(1):94–101. doi: 10.1002/jmd2.12174 (PMC7802624; doi:10.1002/jmd2.12174)

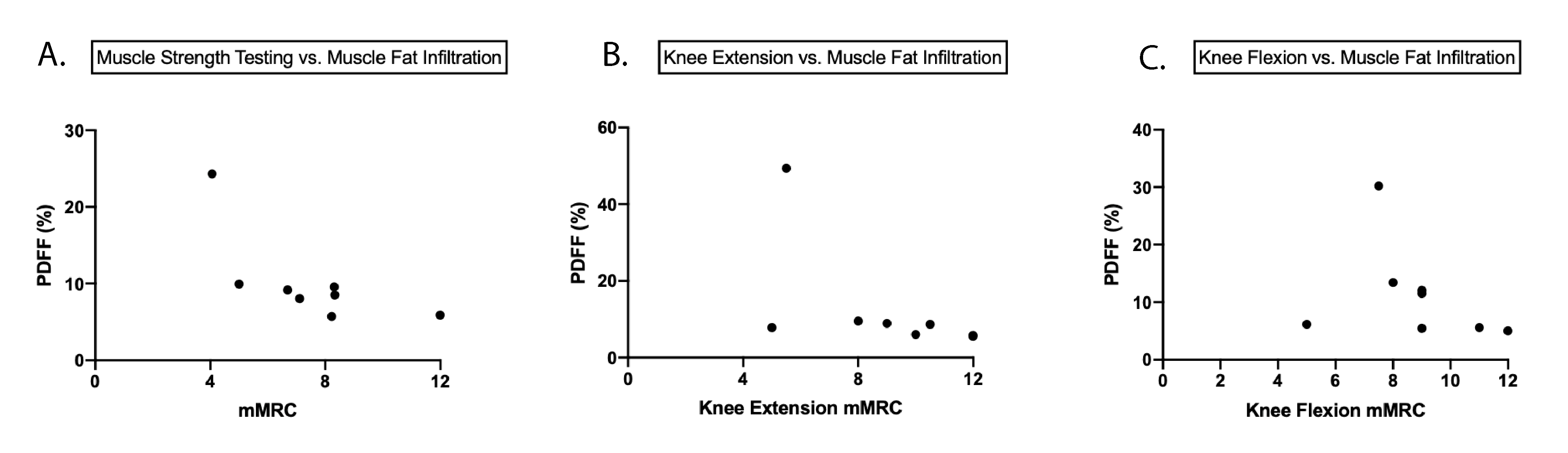

Supplement: Supplementary file 1 — Figure S1: A‐C (left to right): Graphical representations of the PDFF values for each patients' mMRC score. (A) plots the overall average mMRC based on a mean of the distinct strength testing; while (B) and (C) are the mMRC scores of strength testing around a specific joint. PDFF values were calculated based on the muscles involved in the actions performed. PDFF: proton density fat fraction, mMRC: modified manual research council [file JMD2-57-94-s001.tif]

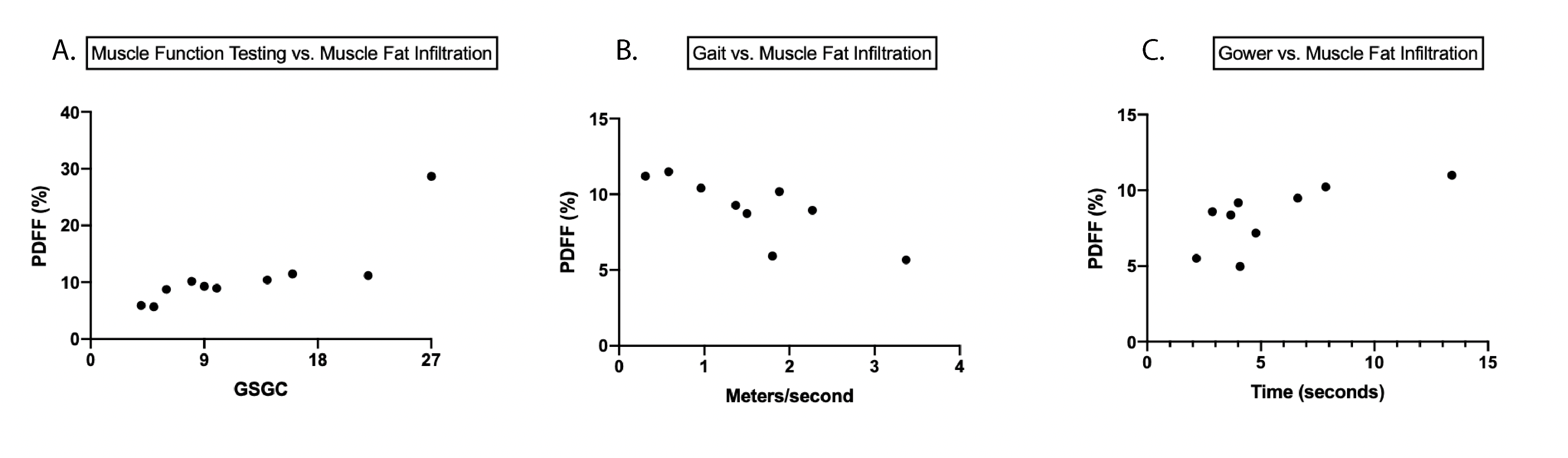

Supplement: Supplementary file 2 — Figure S2: A‐C (left to right): Graphical representations of the PDFF values for each patients' GSGC score. (A) plots the overall average GSGC score; while (B) and (C) are the timed gait testing (meters/second) and gower testing (seconds). PDFF values were calculated based on the muscles involved in the actions performed. PDFF: proton density fat fraction, mMRC: modified manual research council, GSGC: gait, stairs, gower, chair [file JMD2-57-94-s002.tif]
