# Supplementary material for: Quantitative whole‐body magnetic resonance imaging in children with Pompe disease: Clinical tools to evaluate severity of muscle disease
Source: JIMD Rep. 2020 Oct 14;57(1):94–101. doi: 10.1002/jmd2.12174 (PMC7802624; doi:10.1002/jmd2.12174)
Supplement: Supplementary file 3 — Table S1: (a) Muscles involved in manual muscle testing conducted in the study. (b) Muscles involved in the gait, stairs, gowers, chairs assessment used in this study. Table S2: Table describing the demographic information of our subjects. Table S3: Mean PDFF values of specific muscles, IPD and LOPD. [file JMD2-57-94-s003.docx]

Supplementary Tables:

| **Muscles** | **Assist in:** |
| --- | --- |
| Gluteus Maximus | Hip Extension (with flexed knee) |
| Gluteus Maximus, Hamstrings | Hip Extension (with extended knee) |
| Gluteus Medius | Hip Abduction |
| Iliopsoas, Rectus Femoris | Hip Flexion |
| Adductor Magnus, Longus, Brevis | Hip Adduction |
| Hamstrings | Knee Flexion |
| Vastus Medialis, Intermedius, Lateralis,  Rectus Femoris | Knee Extension |
| Anterior Tibialis | Foot/Ankle dorsiflexion |

Table S1 a: Muscles involved in manual muscle testing conducted in the study

| **Muscles** | **Functional assessment:** |
| --- | --- |
| Vastus Medialis, Intermedius, Lateralis  Rectus Femoris  Iliopsoas  Anterior Tibialis  Gluteus Maximus  Gluteus Medius  Thoracic Spinal Extensors  Lumbar Spinal Extensors  Adductor Magnus, Longus, Brevis  Hamstrings | **Gait Speed**  **Gait, Stairs, Gower, Chair (GSGC) Assessment** |
| Gluteus Maximus  Hamstrings  Vastus Medialis, Intermedius, Lateralis  Iliopsoas  Rectus Femoris | **Climb 4 stairs** |
| Gluteus Maximus  Hamstrings  Vastus Medialis, Intermedius, Lateralis  Thoracic Spinal Extensors  Lumbar Spinal Extensors  Gluteus Medius | **Stand from supine** |
| Gluteus Maximus  Hamstrings  Vastus Medialis, Intermedius, Lateralis  Thoracic Spinal Extensors  Lumbar Spinal Extensors  Gluteus Medius  Adductor Magnus, Longus, Brevis | **Stand from sitting** |

Table S1 b: Muscles involved in the Gait, Stairs, Gowers, Chairs assessment used in this study.

| **Pt no.** | **Pompe Type** | **GAA variant (allele 1)** | **GAA variant (allele 2)** | **Ambulatory device?** | **Age at Dx (y)** | **Age at ERT start (y)** | **Age at WBMRI (y)** | **ERT dose at time of WBMRI** |
| --- | --- | --- | --- | --- | --- | --- | --- | --- |
| 1 | IPD | c.1933G>A | c.1933G>A | No | <0.25 | <0.3 | 16 | 30mg/kg w |
| 2 | IPD | c.1293_1312del | c.1716C>G | No | <0.1 | <0.2 | 8 | 40mg/kg w |
| 3 | IPD | c.1933G>A | c.1933G>A | Walker | <0.2 | <0.3 | 13 | 39mg/kg w |
| 4 | IPD | c.2297A>C | c.2297A>C | Wheelchair | <0.6 | <0.7 | 14 | 40mg/kg w |
| 5 | IPD | c.546+2_546+5del | c.1650dup | No | birth | <0.1 | 7 | 20mg/kg eow |
| 6 | LOPD | c.-32-13T>G | c.525del | No | <0.8 | <1.7 | 10 | 40mg/kg w |
| 7 | LOPD | c.-32-13T>G | c.1437+2T>C | No | <8 | <8.5 | 16 | 20mg/kg eow |
| 8 | LOPD | c.-32-13T>G | c.953T>C | No | <13 | <13 | 17 | 40mg/kg eow |
| 9 | LOPD | c.1477C>T | c.1978C>T, and c.2221G>A | No | <6 | <6 | 14 | 40mg/kg w |
| 10 | LOPD | c.-32-13T>C | c.2560C>T | No | <12 | <12 | 12 | 20mg/kg eow |
| 11 | LOPD | c.-32-13T>G | c.2501_2502del | No | <2 | <2.3 | 9 | 20mg/kg eow |

Table S2: Table describing the demographic information of our subjects.

Pt: patient, IPD: Infantile Pompe Disease, LOPD: Late-onset Pompe Disease, Dx: diagnosis, ERT: enzyme replacement therapy, WBMRI: whole-body magnetic resonance imaging, y: years, w: weekly, eow: every other week

| **Muscles** | **IPD** | **LOPD** |
| --- | --- | --- |
| Trapezius | 6.77 | 6.67 |
| Thoracic Extensors | 8.9 | 7.37 |
| Serratus Anterior | 9 | 7.52 |
| Iliopsoas | 9.11 | 8.81 |
| Rhomboids | 9.12 | 7.45 |
| Lumbar Extensors | 9.56 | 5.86 |
| Adductor Muscles | 10.5 | 7.57 |
| Gluteus Medius | 10.8 | 7.41 |
| Anterior Tibialis | 12.1 | 11.1 |
| Hamstrings | 12.8 | 8.95 |
| Gluteus Maximus | 16 | 11 |
| Rectus Femoris | 16.3 | 13.3 |
| Vastus Muscles | 18.5 | 7.76 |

Table S3: Mean PDFF values of specific muscles, IPD and LOPD.

PDFF: proton density fat-fraction, LOPD: late onset Pompe disease, IPD: infantile Pompe disease
